# Changes of Serum Adiponectin Level in Patients with Obstructive Sleep Apnea Hypopnea Syndrome and Its Relationship with Sleep Monitoring Indexes

**DOI:** 10.1155/2024/4071131

**Published:** 2024-03-15

**Authors:** Ji Li, Kejing Zhou, Xing Chen

**Affiliations:** ^1^Department of Otorhinolaryngology, Head and Neck Surgery, The First Affiliated Hospital of Ningbo University, China; ^2^Ningbo Yinzhou Second Hospital, Ningbo, China; ^3^Department of Ophthalmology, Yinzhou Second Hospital, Ningbo, China

## Abstract

**Objective:**

To observe the changes of serum adiponectin (AP) levels in patients with obstructive sleep apnea hypopnea syndrome (OSAHS) and explore the correlation between serum AP and polysomnography (PSG) parameters in patients with OSAHS.

**Methods:**

The data of subjects who underwent PSG at the hospital between January 2021 and December 2022 were collected retrospectively and divided into simple snoring group (AHI < 5 times/h, *n* = 45), mild OSAHS group (5 ≤ AHI < 15 times/h, *n* = 63), moderate OSAHS group (15 ≤ AHI ≤ 30 times/h, *n* = 52), and severe OSAHS group (AHI > 30 times/h, *n* = 60). The general data, PSG indices, and serological indices of the subjects were collected and compared between groups. Pearson correlation analysis and partial correlation analysis were employed to examine the correlation between serum AP level and PSG parameters. Ordered logistic regression was employed to analyze the risk factors influencing the severity of OSAHS. The predictive capability of the serum AP level in determining the occurrence of OSAHS was assessed using ROC. The serum AP levels of subjects with different subtypes of PSG indicators were compared.

**Results:**

In the simple snoring group, mild OSAHS group, moderate OSAHS group, and severe OSAHS group, there were statistically significant differences in microarousal count, MAI, AHI, times of blood oxygen decreased by ≥ 3%, L-SaO_2_, and TS90% among the 4 groups (*P* < 0.05). The level of serum AP was positively correlated with L-SaO_2_ and negatively correlated with the proportion of REM, microarousal count, MAI, AHI, times of blood oxygen decreased by ≥ 3%, TS90%, and LP (*P* < 0.05). High AHI was a risk factor affecting the severity of OSAHS (95% CI: 1.446–4.170). The AUC of serum AP level in diagnosing OSAHS was 0.906 (95% CI: 0.8601–0.9521), and when the Youden Index was 0.678, the sensitivity was 88.9%, and the specificity was 78.9% (*P* < 0.0001). In the population with a high microarousal count, high AHI, and high times of blood oxygen decreased by ≥ 3% and high TS90%, the serum AP level was lower than that in the low-level population (*P* < 0.05). In the population with high L-SaO_2_, the serum AP level was higher than that in low-level population (*P* < 0.05).

**Conclusion:**

The level of serum AP decreased with the increase of the disease severity of patients with OSAHS and demonstrates a significant predictive capability for the occurrence of OSAHS. Monitoring the level of serum AP can effectively forecast the risk of OSAHS. Furthermore, alterations in serum AP levels are associated with both hypoxemia and a heightened frequency of arousal in patients with OSAHS.

## 1. Introduction

Obstructive sleep apnea hypopnea syndrome (OSAHS) is a sleep respiratory disorder characterized by persistent partial upper respiratory tract obstruction and/or intermittent complete obstruction (obstructive apnea), which disrupts normal sleep ventilation and sleep patterns [1]. Epidemiological research indicates that OSAHS is the most prevalent sleep-disordered breathing disorder, with an incidence rate of up to 38% in the adult population, including 13%–33% for males and 6%–19% for females. Furthermore, the prevalence of OSAHS is higher in obese individuals compared to nonobese individuals [2]. There exists a significant correlation between OSAHS and obesity, with a majority of OSAHS patients exhibiting symptoms of obesity. Furthermore, obesity has been identified as the sole reversible factor of importance [3]. Previous research has indicated that obesity, as a risk factor for OSAHS, may be associated with the dysregulation of endogenous hormones and abnormal adipose tissue synthesis [4].

Adiponectin (AP), a recently discovered secretory protein composed of 244 amino acids, is present in various cellular and adipose tissue configurations. AP, an essential regulatory factor in the lipid metabolism and blood glucose homeostasis regulatory network, possesses various functions including blood glucose and lipid reduction, insulin resistance improvement, anti-inflammatory effects, and atherosclerosis antagonism [5]. In a study conducted by Magalang et al. [6], it was observed that the presence of intermittent hypoxia led to a decrease in AP levels when adipocytes were incubated in vitro for 48 hours. This finding suggests that, in addition to obesity, sleep-disordered breathing plays a significant role in the reduction of serum AP levels. In the mouse model of persistent intermittent hypoxemia, a reduction in AP circulation level was observed, indicating a potential association between decreased AP and the occurrence of OSAHS [7].

Previous research has demonstrated that individuals with OSAHS typically exhibit decreased serum AP levels, which are closely linked to obesity. However, the correlation between the severity of OSAHS and AP remains inconclusive, and there is limited research on the correlation between AP and sleep monitoring indexes in OSAHS patients. This study examines the variations in AP levels among patients with OSAHS of different severity and its correlation with polysomnography (PSG) parameters. In addition, the diagnostic value of serum AP for OSAHS and the predictive value of serum LP for the occurrence of OSAHS in a specific population are discussed. The findings aim to offer insights for the prevention and treatment of OSAHS.

## 2. Data and Methods

### 2.1. General Information

The data of subjects who underwent PSG at the hospital between January 2021 and December 2022 were collected retrospectively, included 220 patients. According to the apnea hypopnea index (AHI), the subjects were categorized into four groups: simple snoring group (AHI < 5 times/h, *n* = 45), mild OSAHS group (5 ≤ AHI < 15 times/h, *n* = 63), moderate OSAHS group (15 ≤ AHI ≤ 30 times/h, *n* = 52), and severe OSAHS group (AHI > 30 times/h, *n* = 60). Inclusion criteria are as follows: (1) aged 18 years or older; (2) all patients with OSAHS met the diagnostic criteria of OSAHS [8]; (3) diagnostic criteria of simple snoring: Participants must meet the diagnostic criteria for simple snoring, which includes snoring in different degrees during the night, AHI < 5 times/h, and being asymptomatic during the day; and (4) patient with a normal mental state. Exclusion criteria are as follows: (1) Patient with sleep disorders other than OSAHS; (2) patient with a history of OSAHS treatment; (3) patient used psychotropic drugs and sedative-hypnotic drugs; (4) patients with hepatic and renal insufficiency, patient with malignant tumor and other serious diseases; and (5) patients with serious infections; pregnant or lactating women. This study was approved by the Medical Ethics Committee of the First Affiliated Hospital of Ningbo University.

### 2.2. Research Methods

#### 2.2.1. PSG Monitoring

General characteristics such as sex, age, body mass index (BMI), smoking history, and drinking history of all the subjects were collected. Prior to monitoring, all participants were required to abstain from smoking and alcohol for a period of 48 h. In addition, they were instructed to avoid consuming stimulating foods such as coffee, tea, alcohol, and sleep-interfering drugs. On the day of monitoring, patients were informed about the purpose, significance, and methods of the monitoring process, in order to mitigate potential psychological conditions such as tension and anxiety. Subsequently, all patients underwent monitoring in a tranquil and comfortable environment using the polysomnography system in a designated sleep monitoring room, with a minimum recording duration of 7 h. The monitoring results were automatically analyzed by computers and corrected by sleep experts. The total sleep time (TST), the sleep efficiency (SE), the proportion of rapid eye movement (REM), the average heart rate (AHR), the microarousal count, the microarousal index (MAI), AHI, the times of blood oxygen decreased by ≥ 3%, the lowest oxygen saturation (L-SaO_2_), the mean oxygen saturation (M-SaO_2_), and the time in saturation lower 90% (TS90%) were collected.

#### 2.2.2. Collection of Serological Indices

In the morning following PSG, a fasting peripheral venous blood sample of 5 ml was obtained from the subjects. The serum was collected after undergoing centrifugation at a speed of 3000 r/s for 10 min, which should then be stored at a temperature of −80°C. The levels of serum total cholesterol (TC), triacylglycerol (TG), high-density lipoprotein cholesterol (HDL-C) and low-density lipoprotein cholesterol (LDL-C), orexins (Ox), leptin (LP), and AP were determined.

### 2.3. Statistical Methods

Data analysis was performed using SPSS 26.0. The counting data was quantified by the number of cases, and the *χ*^2^ test was employed to compare between groups. Measurement data that followed a normal distribution were represented by the mean ± standard deviation ($\bar{x}\pm s$), and analysis of variance was used to compare among groups. Measurement data that did not conform to a normal distribution were represented by the M (QR). The Kruskal–Wallis test was employed to compare among groups. Pearson correlation analysis and partial partial correlation analysis were employed to examine the correlation between serum AP level and PSG parameters. Ordered Logistic regression was employed to analyze the risk factors influencing the severity of OSAHS. The predictive capability of serum AP level in determining the occurrence of OSAHS was assessed using the receiver operating characteristic curve (ROC). *P* < 0.05 was statistically significant.

## 3. Results

### 3.1. Comparison of Basic Data between Simple Snoring Group and OSAHS Group

There was no significant difference in general data such as sex, age, BMI, smoking history, and drinking history among the simple snoring group, the mild OSAHS group, the moderate OSAHS group, and the severe OSAHS group (*P* > 0.05) as shown in Table 1.

In the simple snoring group, mild OSAHS group, moderate OSAHS group, and severe OSAHS group, there was no significant difference in the TST, SE, and proportion of REM, AHR, and M-SaO_2_ (*P* > 0.05); there were statistically significant differences in microarousal count, MAI, AHI, and times of blood oxygen decreased by ≥ 3%, L-SaO_2_ and TS90% among the 4 groups (*P* < 0.05). The microarousal count, MAI, AHI, times of blood oxygen decreased by ≥ 3% and TS90% in the OSAHS group were higher than those in the simple snoring group, and the L-SaO_2_ was lower than that in simple snoring group (*P* < 0.05) as shown in Table 2.

There was no significant difference in the levels of TC, TG, HDL-C, LDL-C, and Ox among the simple snoring group, mild OSAHS group, moderate OSAHS group, and severe OSAHS group (*P* > 0.05); there were significant differences in the levels of LP and AP among the 4 groups (*P* < 0.05). The level of LP in the OSAHS group was higher than that in the simple snoring group, and the level of AP was lower than that in the simple snoring group (*P* < 0.05) as shown in Table 3.

### 3.2. Correlation Analysis between Serum AP Level and Clinical Indexes

The level of serum AP was positively correlated with BMI and L-SaO_2_ (*r* = 0.162, *r* = 0.523, *P* < 0.05), and negatively correlated with the proportion of REM, microarousal count, MAI, AHI, times of blood oxygen decreased by ≥ 3%, TS90% and LP (*r* = −0.173, *r* = −0.350, *r* = −0.351, *r* = −0.743, *r* = −0.733, *r* = −0.592, *r* = −0.618, *P* < 0.05). There was no correlation between serum AP level and age, TST, SE, AHR, M-SaO_2_, TC, TG, HDL-C, LDL-C, and Ox (*P* > 0.05) as shown in Table 4.

**Figure 1:**
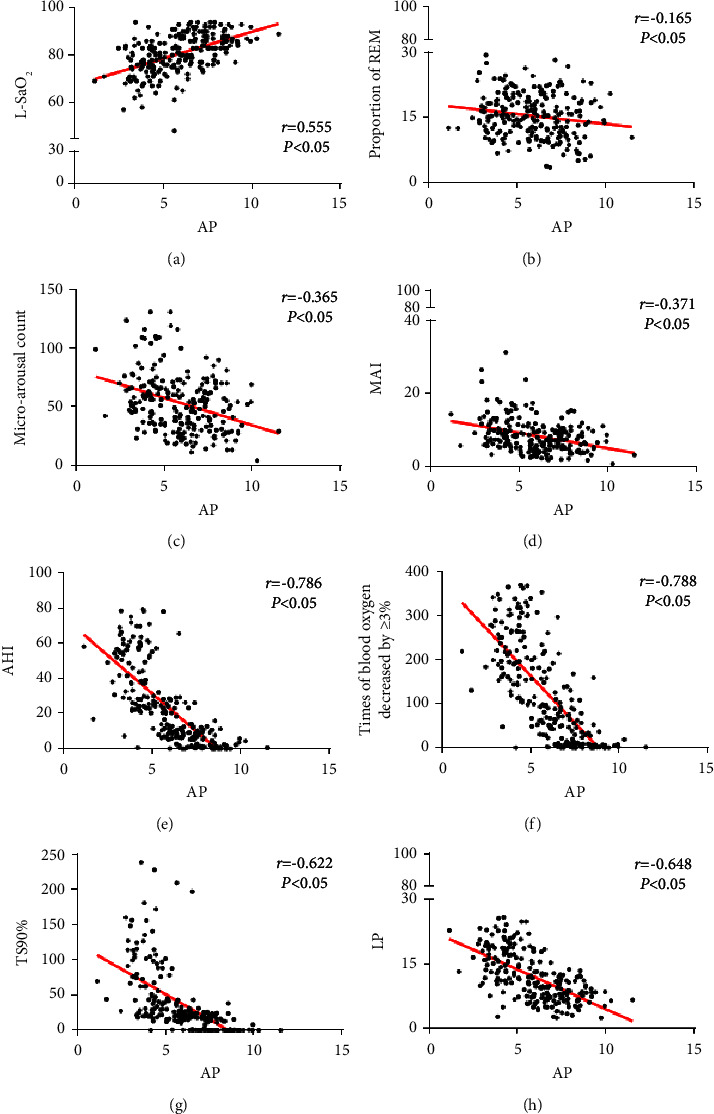
Correlation analysis between serum AP level and clinical indexes. (a) Correlation analysis of serum AP level and L-SaO_2_; (b) correlation analysis of serum AP level and the proportion of REM; (c) correlation analysis of serum AP level and microarousal count; (d) correlation analysis of serum AP level and MAI; (e) correlation analysis of serum AP level and AHI; (f) correlation analysis of serum AP level and times of blood oxygen decreased by ≥ 3%; (g) correlation analysis of serum AP level and the TS90%; (h) correlation analysis of serum AP level and serum LP level.

After controlling BMI, the level of serum AP was positively correlated with L-SaO_2_ (*r* = 0.555, *P* < 0.05), and negatively correlated with the proportion of REM, microarousal count, MAI, AHI, times of blood oxygen decreased by ≥ 3%, TS90%, and LP (*r* = −0.165, *r* = −0.365, *r* = −0.371, *r* = −0.786, *r* = −0.788, *r* = −0.622, *r* = −0.648, *P* < 0.05) as shown in Figure 1.

### 3.3. Analysis of Independent Factors Affecting the Severity of OSAHS Patients

The proportion of REM, microarousal count, MAI, AHI, times of blood oxygen decreased by ≥ 3%, L-SaO_2_, TS90%, LP, and AP were taken as independent variables, and the severity of OSAHS were taken as dependent variables, the ordered logistic regression analysis showed that the high AHI was a risk factors affecting the severity of OSAHS (*P* < 0.05). When AHI was increased by one unit, the change range of OSAHS condition was 2.455 times (95% CI: 1.446–4.170; *χ*^2^ = 11.069, *P* < 0.001) as shown in Tables 5 and 6.

### 3.4. Diagnostic Value Analysis of Serum AP Level for OSAHS

**Figure 2:**
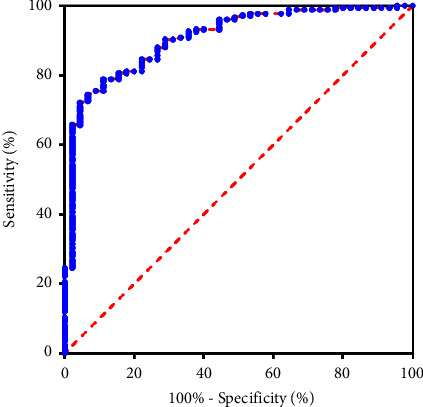
ROC of serum AP level predicting the occurrence of OSAHS.

The area under the curve (AUC) of serum AP level in diagnosing OSAHS was 0.906 (95% CI: 0.8601–0.9521). When the Youden index was 0.678, the sensitivity was 88.9% and the specificity was 78.9% (*P* < 0.0001) as shown in Figure 2.

### 3.5. Comparison of Serum AP Levels in OSAHS Patients with Different PSG Parameters

**Figure 3:**
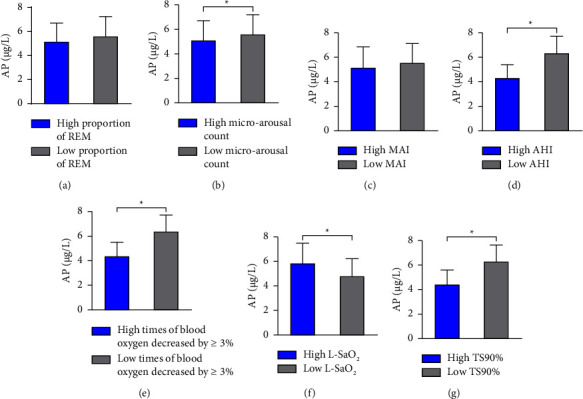
Comparison of serum AP levels in OSAHS patients with different PSG parameters. (a) Comparison of serum AP levels in OSAHS patients with different proportions of REM; (b) comparison of serum AP levels in OSAHS patients with different microarousal count; (c) comparison of serum AP levels in OSAHS patients with different MAI; (d) comparison of serum AP levels in OSAHS patients with different AHI; (e) comparison of serum AP levels in OSAHS patients with different times of blood oxygen decreased by ≥ 3%; (f) comparison of serum AP levels in OSAHS patients with different L-SaO_2_; (g) comparison of serum AP levels in OSAHS patients with different TS90%. *Note*. Compared with the serum AP level of population with low PSG parameter, ^*∗*^*P* < 0.05.

The author further subtype analysis was carried out on different PSG parameters of OSAHS patients (proportion of REM, microarousal count, MAI, AHI, and times of blood oxygen decreased by ≥ 3%, L-SaO_2_, TS90%). The median value was used as the limit for typing. In the population with high microarousal count, high AHI, and high times of blood oxygen decreased by ≥ 3% and high TS90%, the serum AP level was lower than that in the low-level population (*P* < 0.05). In the population with high L-SaO_2_, the serum AP level was higher than that in low-level population (*P* < 0.05). However, there was no significant difference in serum AP level among different proportions of REM and different MAIs (*P* > 0.05) as shown in Table 7 and Figure 3.

### 3.6. Typical Patient Report

**Figure 4:**
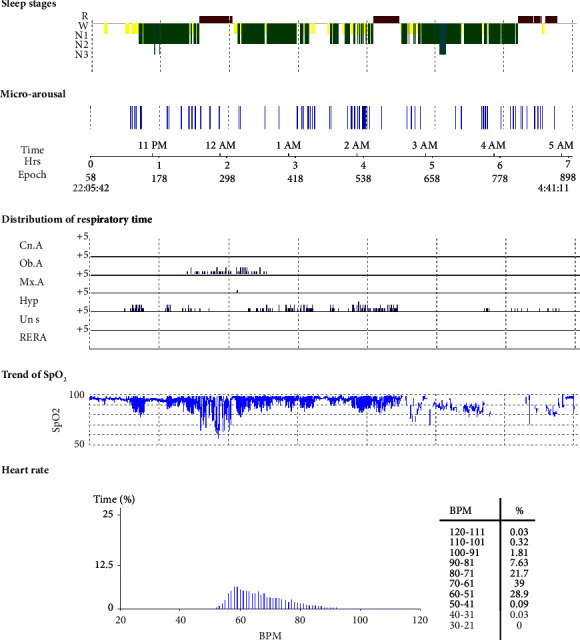
PSG monitoring report of severe OSAHS patients.

A 30-year-old male patient was admitted to the hospital due to symptoms including frequent episodes of dyspnea, wheezing at night, and drowsiness and dizziness during daytime. The patient had a BMI of 30.4 kg/m^2^ and a 10-year alcohol abuse history. PSG monitoring report showed that the patient was consistent with severe OSAHS with severe hypoxemia. Sleep structure: TST of 348.5 min, sleep latency of 11.0 min, REM latency of 83.0 min, SE of 82.2%, microarousal count of 76 times, MAI of 13.1 times/h, sleep stage transition times of 138 times. The proportion of sleep stages: N1 stage accounted for 15.2%, N2 stage accounted for 59.1%, N3 stage accounted for 2.2%, and REM stage accounted for 23.5%. No abnormal manifestations of nocturnal molars and REM phase-related disorders were observed. Respiratory events: AHI of 38.0 times/h, maximum apnea time of 65 s, maximum hypopnea time of 54 s. The heart rate slowed down when respiratory events occurred, and increased after respiratory events, and was accompanied by audible snoring. Blood oxygen status: M-SaO_2_ during sleep of 88%, L-SaO_2_ during sleep of 57%. Times of blood oxygen decreased by ≥ 3% of 279 times, TS90% of 2 h 40 min 12 s. Electrocardiographic events: AHR of 65 bpm, slowest HR of 39 bpm, fastest HR of 94 bpm. Serological detection: TC of 4.6 mmol/L, TG of 1.9 mmol/L, HDL-C of 1.6 mmol/L, LDL-C of 3.0 mmol/L, LP of 21.6 *μ*g/L, OX of 401.7 *μ*g/L, AP of 4.83 *μ*g/L. Patients underwent noninvasive positive airway pressure ventilation, concomitant with weight management, alcohol cessation, and lateral sleeping, in order to ameliorate the condition and enhance sleep quality. After treatment, the symptoms of the patients were better than when they were admitted to hospital. The patient reported that the daytime vitality increased and the symptoms of drowsiness and dizziness were obviously improved as shown in Figure 4.

## 4. Discussion

OSAHS, being the most prevalent sleep respiratory disorder, is characterized by repetitive collapse of the upper respiratory tract during sleep [9]. PSG serves as the “gold standard” for diagnosing OSAHS, as it monitors various physiological changes including electrocardiogram, electroencephalogram, electrooculogram, mandibular electromyography, chest and abdomen movement, blood oxygen saturation, snoring sounds, and others. In addition, PSG monitors the AHI during sleep and considers daytime sleepiness, signs, and complications for a comprehensive diagnosis and severity grade [10, 11].

The results of this study show that the microarousal count, MAI, AHI, times of blood oxygen decreased by ≥ 3%, and TS90% in the OSAHS group were higher than those in simple snoring group, and the L-SaO_2_ was lower than that in simple snoring group. The findings of this study suggest that patients with OSAHS exhibit a disordered sleep structure, characterized by increased microarousal count, MAI, and AHI, as well as decreased L-SaO_2_. These factors are considered to be the primary contributors to the diminished sleep quality and reduced sleep efficiency in OSAHS patients. In addition, this study revealed that with the increase of disease severity of patients with OSAHS, the LP level showed an obvious upward trend and the AP level showed an obvious downward trend. Repeated episodes of hypoxia in patients with OSAHS can induce an upregulation of LP secretion. Concurrently, it can also enhance the expression of inflammatory genes and certain adipokine genes in adipocytes through the activation of NF-kB and HIF-1*α*. This activation leads to an increase in the secretion of various adipokines, while inhibiting the secretion of AP by suppressing the synthesis of AP mRNA [4, 12, 13].

The author employed ordered logistic regression analysis and concluded that a high AHI serves as a risk factor influencing the severity of OSAHS. In comparison to individuals with a low AHI, those with a higher AHI exhibit a more severe of OSAHS. AHI is commonly employed in clinical settings to assess the severity of OSAHS, as it reflects the frequency of nocturnal respiratory events associated with OSAHS. This parameter effectively distinguishes simple snoring from OSAHS and plays an essential role in clinical diagnosis and treatment. Numerous studies have substantiated a significant correlation between weight loss resulting from dietary and lifestyle modifications and the reduction of AHI in patients diagnosed with OSAHS [14–16]. Zeng et al. [17] demonstrated a negative correlation between AP level and AHI in obese patients with OSAHS. These results suggest that sleep apnea and fragmented sleep increase, and whether the hypoxia state of adipocytes directly or indirectly inhibits AP expression remains to be further studied. The author analyzed the value of serum AP level in diagnosing OSAHS. The results showed that the AUC of serum AP level in diagnosing OSAHS was 0.906, the sensitivity was 88.9%, and the specificity was 78.9%. The findings demonstrate that serum AP exhibits significant predictive value in determining the occurrence of OSAHS, suggesting its potential use as an additional monitoring index for diagnosing and treating OSAHS.

Obesity is widely recognized as a crucial pathogenic factor contributing to OSAHS, and weight loss is commonly recommended as part of OSAHS treatment [18]. Wang et al. [19] reported a 30% increase in OSAHS risk for every 10% increase in body weight, potentially attributed to the collapse of the upper respiratory tract resulting from adipose tissue deposition in the throat mucosa. The results of this study show that after controlling BMI, the level of serum AP was positively correlated with L-SaO_2_ and negatively correlated with the proportion of REM, microarousal count, MAI, AHI, times of blood oxygen decreased by ≥ 3%, TS90%, and LP. This finding is consistent with the research conducted by Yoshikawa et al. the study revealed an elevation in plasma AP levels following prolonged continuous positive airway pressure therapy, indicating a potential association between reduced plasma AP levels and oxidative stress induced by OSAHS [20]. The increased inflammatory response of adipocytes in OSAHS patients, potentially caused by obesity and other factors, may inhibit the expression of the APN gene. Moreover, individuals experiencing repeated hypoxia and intermittent apnea induce a state of hypoxia-reoxygenation alternation in the body, resembling ischemia-reperfusion injury, which will aggravate the activation of oxidative stress and the inflammatory response, leading to a significant increase in the expression of TNF-*α* and IL-6. IL-6 and TNF-*α* possess the ability to inhibit the synthesis and secretion of APN [21–23]. Wang et al. [24] also verified that the functional damage caused by hypoxia in OSAHS patients can be counteracted through AP supplementation on chronic intermittent hypoxia model rats. OSAHS has the potential to elevate the level of oxidative stress.

Frequent arousal during sleep is a prevalent manifestation of OSAHS, which can result in sleep fragmentation. The AHI is considered the most reliable indicator for assessing the extent of sleep fragmentation and nocturnal hypoxemia during PSG monitoring [25]. In this study, PSG indexes with differences were categorized, revealing that the serum AP level was lower in individuals with a high microarousal count, high AHI, high times of blood oxygen decreased by ≥ 3%, and high TS90%, compared to the low-level population. However, the opposite trend was observed in the high-level population with L-SaO_2_. Nocturnal hypoxemia and increased arousal times resulting from OSAHS can induce sympathetic nervous system activity and elevate serum catecholamine levels. Isoproterenol has been found to inhibit the expression of the APN gene. Research has demonstrated that *β*-adrenoceptor agonists can have a detrimental impact on AP secretion, with isoproterenol inhibiting the expression of the AP gene in a dose-dependent manner. Moreover, *β*-adrenoceptor agonists can significantly inhibit AP gene expression through the G(S) protein-PKA-dependent pathway, ultimately leading to reduced serum AP levels in patients [26].

This study is limited by its retrospective design, which resulted in a lack of treatment and follow-up information for patients with OSAHS, as well as the absence of a comparative analysis of AP level changes pre- and post-treatment. In addition, the relatively small sample size cannot reflect the entire population of individuals with OSAHS, potentially introducing bias in the analysis. Therefore, future research should aim to validate and analyze these results through multicenter studies with larger sample sizes.

In conclusion, the level of serum AP decreased with the increase of the disease severity of patients with OSAHS and demonstrates a significant predictive capability for the occurrence of OSAHS. Monitoring the level of serum AP can effectively forecast the risk of OSAHS. Furthermore, alterations in serum AP levels are associated with both hypoxemia and heightened frequency of arousal in patients with OSAHS.

## Figures and Tables

**Table 1:** Comparison of basic data between the simple snoring group and OSAHS group ($\bar{x}\pm s$, %).

Item	Simple snoring group (*n* = 45)	Mild OSAHS group (*n* = 63)	Moderate OSAHS group (*n* = 52)	Severe OSAHS group (*n* = 60)	*χ* ^2^/*F* value	*P* value
Sex					1.214	0.750
Male	34 (75.56%)	47 (74.60%)	37 (71.15%)	48 (80.00%)		
Female	11 (24.44%)	16 (25.40%)	15 (28.85%)	12 (20.00%)		
Age (years)	45.47 ± 9.12	46.35 ± 9.70	46.81 ± 8.88	46.55 ± 10.57	0.173	0.914
BMI (kg/m^2^)	26.22 ± 2.77	26.26 ± 3.18	27.11 ± 3.55	27.48 ± 2.60	2.383	0.070
Smoking history					0.680	0.878
With	17 (37.78%)	22 (34.92%)	21 (40.38%)	25 (41.67%)		
Without	28 (62.22%)	41 (65.08%)	31 (59.62%)	35 (58.33%)		
Drinking history					1.548	0.671
With	16 (35.56%)	25 (39.68%)	23 (44.23%)	28 (46.67%)		
Without	29 (64.44%)	38 (60.32%)	29 (55.77%)	32 (53.33%)		

**Table 2:** Comparison of PSG parameters between the simple snoring group and OSAHS group ($\bar{x}\pm s$).

Item	Simple snoring group (*n* = 45)	Mild OSAHS group (*n* = 63)	Moderate OSAHS group (*n* = 52)	Severe OSAHS group (*n* = 60)	*F*/*H* value	*P* value
TST (min)	382.76 ± 104.80	408.54 ± 67.61	401.46 ± 24.34	377.18 ± 78.38	2.402	0.069
SE (%)	69.40 ± 14.05	73.88 ± 7.03	71.74 ± 7.68	70.39 ± 8.94	2.339	0.074
Proportion of REM	14.46 ± 5.12	14.60 ± 5.13	15.76 ± 5.42	16.74 ± 4.39	2.573	0.055
AHR (bpm)	62.51 ± 4.61	60.68 ± 4.29	62.10 ± 6.77	63.03 ± 4.70	2.334	0.075
Microarousal count (times)	34 (23.5)	46 (32)	58.5 (31.75)	62.5 (29.5)	41.712	<0.0001
MAI (times/h)	5.7 (3.7)	7 (4.9)	8.95 (5.02)	9.9 (6.42)	38.396	<0.0001
AHI (times/h)	0.8 (1.6)	9 (4.6)	24.45 (5.85)	56.2 (18.57)	204.678	<0.0001
Times of blood oxygen decreased by ≥ 3%(times)	3 (6.5)	51 (46)	151 (73.5)	274 (97.5)	191.271	<0.0001
L-SaO_2_ (%)	87.29 ± 4.27	84.67 ± 5.90	77.38 ± 6.54	74.25 ± 7.69	50.175	<0.0001
M-SaO_2_ (%)	95.22 ± 1.35	94.52 ± 2.18	94.79 ± 0.96	94.12 ± 3.33	2.246	0.084
TS90% (min)	0.20 ± 0.33	18.14 ± 5.32	28.44 ± 9.35	96.16 ± 51.35	132.103	<0.0001

**Table 3:** Comparison of serological indices between the simple snoring group and OSAHS group ($\bar{x}\pm s$).

Item	Simple snoring group (*n* = 45)	Mild OSAHS group (*n* = 63)	Moderate OSAHS group (*n* = 52)	Severe OSAHS group (*n* = 60)	*F* value	*P* value
TC (mmol/L)	4.59 ± 0.39	4.64 ± 0.35	4.69 ± 0.40	4.69 ± 0.44	0.682	0.564
TG (mmol/L)	1.69 ± 0.26	1.71 ± 0.27	1.72 ± 0.28	1.75 ± 0.31	0.374	0.772
HDL-C (mmol/L)	1.36 ± 0.19	1.39 ± 0.25	1.37 ± 0.25	1.32 ± 0.26	1.038	0.377
LDL-C (mmol/L)	2.79 ± 0.44	2.80 ± 0.38	2.82 ± 0.49	2.85 ± 0.40	0.197	0.898
Ox (*μ*g/L)	382.65 ± 161.18	390.45 ± 172.03	399.32 ± 154.38	413.14 ± 161.50	0.350	0.789
LP (*μ*g/L)	7.26 ± 2.55	8.01 ± 2.35	14.56 ± 4.14	17.62 ± 4.30	117.760	<0.0001
AP (*μ*g/L)	8.06 ± 1.26	6.84 ± 1.04	5.21 ± 1.15	3.92 ± 0.86	149.685	<0.0001

**Table 4:** Correlation analysis between serum AP level and clinical indexes.

Item	AP		
	*r* value	*R* ^2^ value	*P* value
Age	0.116	0.014	0.085
BMI	0.162	0.026	0.016
TST	0.071	0.005	0.293
SE	0.041	0.002	0.549
Proportion of REM	−0.173	0.030	0.010
AHR	−0.025	0.001	0.717
Microarousal count	−0.350	0.123	<0.0001
MAI	−0.351	0.124	<0.0001
AHI	−0.743	0.551	<0.0001
Times of blood oxygen decreased by ≥ 3%	−0.733	0.537	<0.0001
L-SaO_2_	0.523	0.273	<0.0001
M-SaO_2_	0.122	0.015	0.070
TS90%	−0.592	0.350	<0.0001
TC	−0.075	0.006	0.266
TG	−0.102	0.010	0.131
HDL-C	0.030	0.001	0.654
LDL-C	0.023	0.001	0.735
LP	−0.618	0.382	<0.0001
Ox	−0.076	0.006	0.263

**Table 5:** Assignment of ordered logistic regression analysis.

Variable	Item	Assignment
Dependent variable	Severity of OSAHS	Simple snoring group = 1, mild OSAHS = 2, moderate OSAHS = 3, severe OSAHS = 4

Independent variable	Proportion of REM	Measured value
	Microarousal count	Measured value
	MAI	Measured value
	AHI	Measured value
	Times of blood oxygen decreased by ≥ 3%	Measured value
	L-SaO_2_	Measured value
	TS90%	Measured value
	LP	Measured value
	AP	Measured value

**Table 6:** Ordered logistic regression analysis of risk factors affecting the severity of OSAHS.

Item	*β*	SE	Wald *χ*^2^	*P*	OR	95% CI	
						Lower limit	Upper limit
Proportion of REM	0.003	0.148	0.000	0.982	1.003	0.751	1.340
Microarousal count	0.027	0.063	0.187	0.665	1.027	0.908	1.162
MAI	−0.114	0.449	0.065	0.800	0.892	0.370	2.153
AHI	0.898	0.270	11.069	0.001	2.455	1.446	4.170
Times of blood oxygen decreased by ≥ 3%	0.007	0.020	0.107	0.744	1.007	0.968	1.047
L-SaO_2_	−0.061	0.105	0.346	0.556	0.941	0.766	1.154
TS90%	0.148	0.082	3.259	0.071	1.160	0.987	1.362
LP	−0.001	0.214	0.000	0.996	0.999	0.657	1.519
AP	−0.377	0.469	0.647	0.421	0.686	0.274	1.718

**Table 7:** Comparison of serum AP levels in OSAHS patients with different PSG parameters ($\bar{x}\pm s$).

PSG parameter	Subtype	Number of cases	AP (*μ*g/L)	*t* value	*P* value
Proportion of REM	High level	88	5.16 ± 1.51	1.625	0.106
	Low level	87	5.55 ± 1.67		

Microarousal count	High level	91	5.11 ± 1.59	2.132	0.034
	Low level	84	5.62 ± 1.58		

MAI	High level	88	5.16 ± 1.63	5.162	0.110
	Low level	87	5.55 ± 1.56		

AHI	High level	87	4.32 ± 1.09	11.129	<0.001
	Low level	88	6.38 ± 1.34		

Times of blood oxygen decreased by ≥ 3%	High level	88	4.36 ± 1.15	10.668	<0.001
	Low level	87	6.37 ± 1.33		

L-SaO_2_	High level	90	5.88 ± 1.58	4.716	<0.001
	Low level	85	4.80 ± 1.44		

TS90%	High level	88	4.42 ± 1.22	9.662	<0.001
	Low level	87	6.30 ± 1.36		

## Data Availability

The data that support the findings of this study are available from the corresponding author upon reasonable request.
